# Magnitude and Determinants of Postnatal Mothers' Knowledge of Essential Newborn Care at Home in Rural Ethiopia

**DOI:** 10.3389/fped.2022.860094

**Published:** 2022-04-29

**Authors:** Tamirat Getachew, Merga Dheresa, Addis Eyeberu, Bikila Balis, Tesfaye Assebe Yadeta

**Affiliations:** School of Nursing and Midwifery, College of Health and Medical Sciences, Haramaya University, Harar, Ethiopia

**Keywords:** essential newborn care, knowledge, postnatal mothers, newborn, home delivery

## Abstract

**Introduction:**

Globally, nearly three million children die in the neonatal period. Although there is scant information about rural mothers, the enhancement of mothers' knowledge and skills toward essential newborn care (ENC) is a vital aspect in the reduction of newborn illness and mortality. Thus, this study aimed to assess the magnitude and determinants of mothers' knowledge of ENC.

**Methods:**

A community-based cross-sectional study was conducted among recently delivered women using a multistage sampling method in Chole woreda. Data were collected *via* face-to-face interviews. A multivariate logistic regression model was used to identify the determinant factors with the level of knowledge. Odds ratios with a 95% confidence interval was used to describe association and significance was determined at a *P*-value < 0.05.

**Results:**

Data from 510 mothers were employed for analysis. Overall, 33.5% (95% CI: 29.4, 37.6) of the mothers had good knowledge of ENC. Antenatal care (ANC) visits [AOR: 2.42; 95% CI: (1.50, 3.88)], counseled about ENC during ANC [AOR: 5.71; 95% CI: (2.44, 13.39)], delivery at health institutions [AOR: 2.41; 95% CI: (1.30, 4.46)], religion [AOR 1.99, 95% CI: (1.25, 3.16)], and educational level [AOR = 1.64 95% CI: (1.10, 2.51)] were significantly associated with knowledge of ENC. About 74, 75, and 41% of mothers practiced appropriate cord care, breastfeeding, and thermal care, respectively.

**Conclusion:**

Three out of 10 mothers had a good level of knowledge of ENC. Knowledge gaps identified pertained to cord care, breastfeeding, and thermal care. There is opportunity to enhance maternal knowledge of ENC through improving access to ANC and institutional delivery.

## Introduction

Children are most commonly vulnerable to disease, injury, and death during the neonatal period (1). Globally, nearly three million children lose their life before celebrating 1 month of life. Almost 99% of neonatal mortality occurs in low-income nations, including Ethiopia, where health care systems are weak and most of the mothers deliver at home, attended by untrained birth attendants (2, 3). Applying appropriate health interventions that are accessible, reasonable, and tolerable to mothers may prevent most of these neonatal deaths in low-income countries (4).

The World Health Organization (WHO) declared essential newborn care (ENC) as a comprehensive approach to advance the health of newborns and mothers through interventions throughout the perinatal period (during pregnancy, childbirth, and postnatal period) (1, 5). ENC comprises instant drying and covering of newborns following birth, starting skin-to-skin contact, hygienic cord care, eye care, immunization, sooner initiation of breastfeeding, and late washing. Despite the importance of ENC, its enactment was not satisfactory, especially among those delivered at home in Ethiopia (6, 7).

Globally, most neonatal deaths can be averted through standing maternal and child health curriculum which are associated to clean cord care for the prevention of sepsis, regulating temperature, and early initiation of breastfeeding (2, 8). In addition to skill enhancement and equipping health professionals, enhancing the knowledge of mothers regarding ENC is very critical to increasing the quality of ENC. Knowledge gaps and durable cultural principles influence newborn survival at the moment the neonate is at home with the mother. Therefore, the enhancement of mothers' knowledge and skills toward ENC are vital aspects to sustain life, growth, and development of neonates and reducing neonatal morbidity and mortality (9).

Evidence from research carried out in Mekelle, Ethiopia indicates that only one-third of mothers were knowledgeable regarding ENC. Good knowledge (mothers who responded correctly to at least 75% of questions) of ENC showed association with mothers who were educated, counseled during childbirth and postnatally, were knowledgeable of newborn care, and had good knowledge of newborn signs of illness (10).

However, neonatal death is still high, and the Ethiopian government has implemented various health interventions including health care provider training, improving referral systems, integrating health services, applying packages of the Health Extension Program, and regular vaccination (11). However, most women still give birth at home with untrained birth assistants, which may promote the exercise of harmful traditional practices (12). Little emphasis was given to newborn care until the placenta is delivered. This came with delays in drying or covering and delayed skin-to-skin contact with frequent positioning slightly away from the mother's side. Inappropriate knowledge of mothers and caregivers during this period could decrease the quality of newborn care, which may threaten newborn well-being, possibly increasing neonatal mortality (13).

Although a mother's knowledge of ENC is highly effective in the reduction of neonatal mortality and has been broadly encouraged, information on women's knowledge and its determinant factors toward ENC in a study setting is inadequate. Moreover, most studies have focused on health professionals' knowledge of ENC and on scanty evidence about postnatal women's knowledge of ENC in Ethiopia, particularly in rural settings, where home delivery is high. Thus, this article aimed to assess the magnitude and determinants of mothers' knowledge of ENC in rural Ethiopia.

## Methods and Materials

### Study Period and Setting

This survey was studied at Chole woreda from March 1 to 30 in 2019. The administrative center for Chole woreda is Chole town. There are 4 health centers, 18 health posts, 10 private clinics, and eight pharmacy shops in the woreda. Each health center serves a population of 15,000–25,000 and health posts are attached to each health center (each health post serves 3,000–5,000 people in the woreda). Maternal and child health care services, including antenatal care (ANC), postnatal care (PNC), family planning, and immunization services, were available in the facilities free-of-fee-services. A total of 20 kebeles represented the woreda. The annual report in 2017 of the Chole health office indicated that the health coverage of the woreda attained 57.3% (14).

### Study Design and Population

This is a cross-sectional study conducted at the community level among postnatal mothers who delivered within 6 months before the study period. Postnatal mothers who delivered in the past 6 months at Chole woreda but were incapable to communicate because of serious illness or impaired cognition at the time of the data collection were not included in the study.

### Sample Size Determination and Sampling Procedure

The sample size was computed using a single population proportion formula by assuming a confidence level of 95% = 1.96, a margin of error (d) = 5%, design effect = 1.5, and prevalence of postnatal mothers' knowledge of ENC (*P* = 31%) in accordance to a study conducted in southern Ethiopia in 2017 (15). It also included a 5% non-response rate. Finally, 520 postnatal mothers were included. A multi-stage cluster sampling method was used to select postnatal mothers. Chole woreda has 20 kebeles (the smallest administrative unit; 16 rural and four urban), five and two kebeles from the rural and urban areas, respectively, were included through a simple random sampling technique. The final sample size was allocated to each kebele proportional to the number of their population. Finally, study participants were randomly recruited from the sampling frame found from records of health extension workers of the kebele. All eligible study participants were interviewed until the desired sample size was achieved.

### Data Collection Methods

The data were collected using an interviewer-administered structured questionnaire adapted from previous studies (10, 16–18). The questionnaire included socio-demographic, obstetrics, and reproductive characteristics, knowledge about essential newborn care, and exposure to counseling during the postnatal period. Seven trained health professionals collected the data under the supervision of three health professionals. A face-to-face interview was conducted among volunteer mothers through structured and pretested questionnaires.

### Measurement and Operational Definitions

Some of the questions on several features of newborn care included practice regarding immunization, thermal care, breastfeeding, cleanliness, and umbilical cord care.

Knowledge level was assessed based on questions on various aspects of essential newborn care (e.g., knowledge about immunization, thermal care, breastfeeding, and umbilical cord care were assessed). The values were coded as 1 = Correct response and 0 = Incorrect response. Finally, based on the median score, a composite variable from these questions was generated to categorize mothers as having “Good/poor knowledge.”

#### Good Knowledge

Those postnatal mothers who were able to answer the knowledge questions above or equal to the median (17).

#### Poor Knowledge

Those postnatal mothers who were able to answer the knowledge questions below the median (17).

**Essential newborn care** refers to the postnatal mothers' care for newborns, which includes early breastfeeding, cord care, eye care, immunization, neonatal danger signs, and thermal care (19).

**Safe cord care** refers to the maintenance of the umbilical cord. Particularly, ensuring that the cord clean and dry without application of any substance on the cord stump except for medically indicated medications, such as chlorhexidine (10).

**Optimum thermal care** includes wrapping the newborn in a clean and dry cloth and the delay in bathing for 24 h to avoid hypothermia (10).

**Early breastfeeding** is the act of immediately starting breastfeeding within the first hour of birth without pre-lacteals and feeding the child with colostrum (10).

### Data Quality Control

A pretest among 10% of the computed sample size was carried out to test the quality of the questionnaires. Each question under the questionnaire was checked for completeness and consistency. The questionnaire was translated into Amharic and Afan Oromo languages (language spoken by the local community). The data collectors and supervisors were trained. The collected data was double data entered into Epi Data version 3.1 software.

### Data Processing and Analysis

Descriptive analysis by statistical package for social sciences (SPSS) version 20 stat calc was presented with a proportion of 95% CI. Continued variables were described using a median and interquartile range. The results were shown using frequencies, percentages, tables, and figures. A chi-square assumption was checked before fitting the model. The Hosmer-Lemeshow statistic and Omnibus tests were checked to assure the model's goodness of fit. A binary logistic regression model was utilized to detect the association between determinants and the outcome variable. All variables with *P* ≤ 0.25 in the bivariate analysis were exported to multivariate analysis to control all possible confounders. The degree of a statistical association between determinants and outcome variables was assessed by using an odds ratio at a 95% level of confidence. Statistical significance was stated at a *P*-value < 0.05.

### Ethical Considerations

The Ethical clearance was granted by the College of Health and Medical Sciences, Institutional Health Research Ethics Review Committee (HU-IHRERC) of Haramaya University. Additionally, an official letter that allows us to conduct this study was obtained from the woreda health bureau and governmental officials. After explaining the purpose of the study and their right, informed and voluntary signed consent was obtained from each study participant.

## Results

### Basic Characteristics of Mothers

From the total (*n* = 520) sample size, 510 mothers were employed for the analysis, yielding a 98.1% of response rate. The median (IQR) age of the mothers and their neonates was 28.48 (4.68) years and 2.7 (1.5) months, respectively. The majority of the mothers were married 467 (91.6%) and were rural residents 333 (65.3%; Table 1).

**Table 1:** Socio-demographic characteristics of mothers who delivered in the past 6 months in Chole woreda, Ethiopia in 2019 (*n* = 510).

**Variables**	**Categories**	**Frequency**	**Percent**
Residence	Urban	177	34.7
	Rural	333	65.3
Age of mother	15–24	105	20.6
	25–34	340	66.7
	35–44	65	12.7
Educational level	Primary and below	178	34.9
	Secondary and above	332	65.1
Marital status	Single	9	1.8
	Married	467	91.6
	Divorce/separated	27	5.3
	Widowed	7	1.4
Husbands educational level	Primary and below	273	58.5
	Secondary and above	194	41.5
Religion	Orthodox	372	72.7
	Muslim	113	22.4
	Protestant	25	4.9
Ethnicity	Oromo	253	49.6
	Amhara	255	50.0
	Tigre	2	0.4
Sex of child	Male	269	52.7
	Female	241	47.3
Occupation	Governmental employee	22	4.3
	Private employee	28	5.5
	Housewife	353	69.2
	Merchant	29	5.7
	Farmer	67	13.1
	Other^*^	11	2.2

* *Other-student and daily laborer*.

### Obstetrics Services and Reproductive Health Characteristics

Of the total respondents, about 363 (71.2%) of the mothers were multiparous and 63 (87.6%) of respondents had at least one prior miscarriage. Among mothers who had ANC (483), 237 (49.1%) of them had four or more visits to hospitals or clinics and the majority (392; 76.9%) were visited by Midwives/Nurse followed by health extension workers (HEWs; 84, 16.4%). Regarding the place of delivery, 134 (26.3%) of the mothers gave birth at home. Vaginal delivery accounted for 487 (95.5%) of births, while instrumental delivery and caesarian section accounted for 12 (2.9%) and 8 (1.6%), respectively (Table 2).

**Table 2:** Obstetrics and reproductive health characteristics respondents in 2020 (*n* = 510).

**Variable**	**Categories**	**frequency**	**Percent**
Para	1	112	22.0
	2–4	363	71.2
	≥5	35	6.9
Abortion history	Yes	63	12.4
	No	447	87.6
ANC	Yes	483	94.7
	No	27	5.3
No. of ANC visit	3 or fewer visits	246	50.9
	4 or more visits	237	49.1
ANC seen by	Physician	7	6.7
	Midwifery/Nurses	392	76.9
	HEWs	84	16.4
Got ANC counseling	Yes	364	75.4
	No	119	24.6
Delivery place	Health institution	376	73.7
	Home	134	26.3
Assisted by	Health professional	376	73.7
	TBA	54	10.6
	TTBA	68	13.3
	Relative (friend)	12	2.4
Immediate PNC	Yes	381	74.7
	No	129	25.3
Care-giver during PNC	Midwife/nurse	325	85.3
	HEWs	47	12.3
	Other^*^	9	2.4

* *Other**-** physician and health officer*. *TBA, traditional birth attendant; TTBA, trained traditional birth attendant*.

### The Relationship Between the Number of ANC Visits and Home Delivery

**Figure 1:**
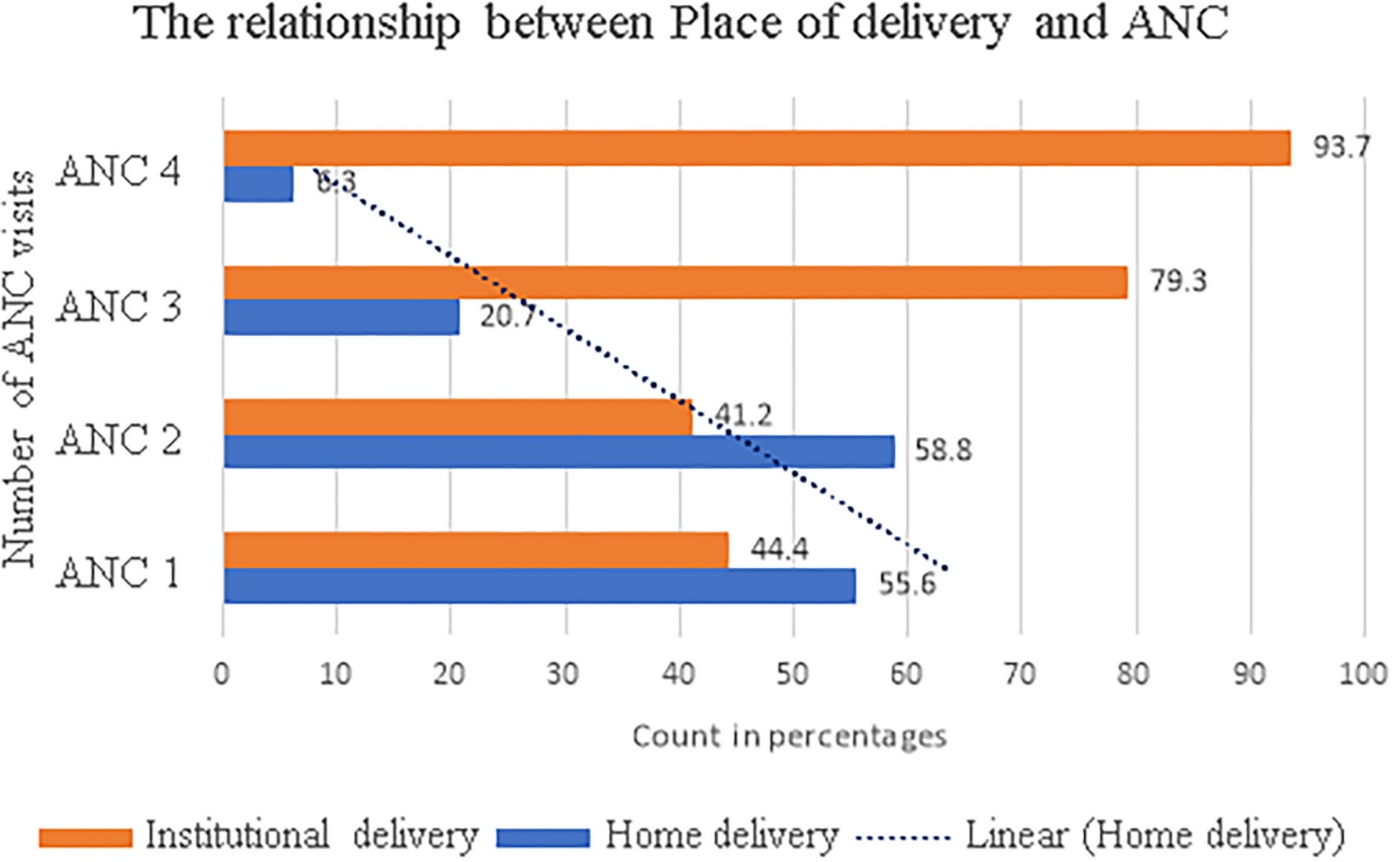
The association between place of delivery and antenatal care (ANC) visit.

Of 483 (94.7%) participants who had ANC visits, 375 (77.6%) mothers delivered at health institutions, and 108 (22.4%) delivered at home. The study revealed that having an ANC visit was protective to attending the subsequent home delivery (Figure 1). Almost 99% of mothers who had no ANC visits delivered at home.

### Exposure to Counseling During Antenatal Care (ANC) Visit

Among the total mothers (483; 94.7%) who had at least one ANC follow-up, only 364 (75.4%) respondents got counseling during their ANC visits. The most commonly attended counseling during ANC visits were counseling about family planning 170 (46.7%), maternal nutrition 143 (39.3%), and breastfeeding 136 (37.4%). On the other hand, counseling on danger signs of pregnancy, immunization, ENC, and neonatal danger signs were poorly addressed areas in all counseling during ANC, in which only 106 (29.1%), 98 (26.9%), 90 (24.7%), and 21 (5.8%) of mothers were counseled respectively.

### Exposure to Counseling During the Postnatal (PNC) Period

None of the respondents had completed all three WHO-recommended PNC visits. However, 381 (74.7%) of respondents had their first or immediate PNC visits, and only 138 (36.2%) of respondents received PNC counseling. During the postnatal visit, the least received counseling was ENC (13.6%) and neonatal danger signs (5%) counseling, respectively (Table 3).

**Table 3:** The type of counseling received by respondents during the postnatal care (PNC) visit at Chole woreda, Ethiopia in 2019 (*n* = 138).

**Type of counseling received**	**frequency**	**Percentage (%)**
**during PNC visit**		
Immunizations	102	73.9
Family planning	97	70.3
Childbirth complications	43	31.2
Nutritional advice	67	48.6
Breastfeeding	114	82.6
Essential newborn care	19	13.6
Neonatal danger signs	7	5
Postnatal care	40	29.1

### Knowledge About Essential Newborn Care (ENC)

Most of the respondents (88.3%) knew at least one component of ENC. However, about 33.5% (95% CI: 29.4, 37.6) of the study participants had good knowledge of ENC and responded above the median score (>4), while 339 (66.5%) had poor knowledge about ENC and responded below the median score (<4). About 75% of respondents think that the newborn baby should not be nursed in a room separated from his/her mother after delivery.

### Cord Care

The majority (377; 73.9%) of the respondents practiced safe cord care. Different instruments were used to tie a cord, of which 250 (49%) stated they used a sterile cord tie, 80 (15.7%) stated unsterile materials were used, and 48 (9.8%) of them did not know the material used. Regarding materials applied on the cord, 298 (58.4%), 34 (6.7%), and 178 (34.9%) stated that they applied nothing, anti-septic solution, and butter, respectively. From the total respondents, 388 (76.1%) have reported that they would go to a health facility if a sign of cord infection was seen. On the other hand, 102 (20%) of women would give home remedies, while 20 (3.9%) of them stated that they would delay until it heals by itself.

### Breastfeeding

When participants were asked about breastfeeding, 386 (75.7%) specified that breastfeeding initiation time was within 1 h after delivery, and 124 (24.3%) of them stated breastfeeding should be initiated after 1 h. Only 203 (39.8%) mothers feed colostrum to their newborn, while the rest do not fed the newborn colostrum due to lack of awareness. Additionally, 350 (68.6%) stated that they exclusively feed their newborns until the first 6 months, while 301 (59%) reported that newborns should exclusively breastfeed until 6 months.

### Thermal Care and Immunization

Of the total respondents, 209 (41%) initiated bathing within 24 h and 301 (59%) initiated bathing after 24 h of delivery. Approximately 422 (82.7%) mothers used a cape to cover the head of the newborn, and approximately 122 (23.9%) study participants exercised skin-to-skin contact with the mother immediately after delivery. From 203 (39.8%) mothers who knew that the newborn needed to be vaccinated at birth, only 27 (5.3%) of respondents immunized their newborn immediately after delivery.

### Determinants of Mothers' Knowledge of Essential Newborn Care

To detect independent determinant factors of ENC, nine variables from socio-demographic, reproductive, and obstetric characteristics and informational related factors having a *P*-value of ≤ 0.25 on bivariate analysis were fitted into the final model. The multivariable logistic regression analysis revealed that good ENC knowledge level among mothers is significantly associated with the number of ANC visits, place of delivery, mother's religion, and educational status.

Antenatal care visit completion was found to be one of the significant determinant factors of a mother's good knowledge of ENC. Mothers who had four or more ANC visits were 2.42 times more likely to have good knowledge of ENC compared to mothers who had less than four ANC visits [AOR: 2.42; 95% CI: (1.50, 3.88)]. The odds of good knowledge were higher among mothers who get counseled about ENC during ANC [AOR: 5.71; 95% CI: (2.44, 13.39)]. The odds of knowing about ENC was 2.41 among mothers who gave birth at health institutions [AOR: 2.41; 95% CI: (1.30, 4.46)] compared to mothers who gave birth at home.

Mothers who are Christian were almost two times more likely to have good knowledge of ENC compared to those whose religion was Muslim [AOR 1.99, 95% CI: (1.25, 3.16)]. Mothers who attained secondary and above educational levels were 1.64 times [AOR= 1.64 95%CI: (1.10, 2.51)] more probable to have good knowledge of ENC compared to mothers who attain primary and lower education levels (Table 4). On the other hand, the age of the mother, parity, partner educational status, source of information, and history of abortion were not significantly associated with maternal knowledge of ENC.

**Table 4:** Bivariate and multivariate logistic regression for determinants of mothers' knowledge of essential newborn care in Chole woreda, Ethiopia in 2019 (*n* = 510).

**Variables**	**Level of knowledge of ENC**		**Odds ratios**	
	**Poor frequency (%)**	**Good frequency (%)**	**COR (95% CI)**	**AOR (95% CI)**
**Residence**				
Rural	242 (72.7)	91 (27.3)	1	1
Urban	97 (54.8)	80 (45.2)	2.19 (1.50, 3.21) ^**^	1.37 (0.88, 2.16)
**Maternal education level**				
Secondary and above Primary and below	152 (67.9)	72 (32.1)	1.12 (0.77, 1.62)	1.64 (1.07, 2.51) ^*^
	187 (65.4)	99 (34.6)	1	1
**Partner's education level**				
Secondary and above Primary and below	29 (85.9)	5 (14.1)	3.11 (1.18, 8.17) ^*^	4.85 (0.67, 35.07)
	310 (65.1)	166 (34.9)	1	1
**Religion**				
Christian	283 (71.3)	114 (28.7)	1	1
Muslim	56 (49.6)	57 (50.4)	2.53 (1.65, 3.88) ^**^	1.99 (1.25, 3.16) ^*^
**Number of ANC**				
1–3	191 (77.6)	55 (22.4)	1	1
4 and above	121 (51.1)	116 (48.9)	3.33 (2.25, 4.93) ^**^	2.42 (1.50, 3.88) ^**^
**Counseled during ANC**				
No	90 (75.6)	29 (24.4)	1	1
Yes	222 (61.0)	142 (39)	1.99 (1.24, 3.17) ^*^	5.71 (2.44, 13.39) ^**^
**Marital status**				
Currently unmarried	34 (79.1)	9 (20.9)	1	1
Currently married	305 (65.3)	162 (34.7)	2.01 (0.94, 4.29)	2.85 (0.52, 15.61)
**Place of delivery**				
Home	117 (87.3)	17 (12.7)	1	1
Health institution	222 (59.0)	154 (41.0)	4.77 (2.76, 8.26) ^**^	2.41 (1.30, 4.46) ^*^
**PNC visit**				
No	290 (70.6)	121 (29.4)	1	1
Yes	49 (49.5)	50 (50.5)	2.45 (1.56, 3.83) ^**^	1.28 (0.77, 2.14)

* *Significant with P < 0.05 and*

** *Significant with P < 0.001*.

*PNC, postnatal care; CI, confidence interval; COR, crude odds ratio; AOR, adjusted odds ratio; ANC, antenatal care*.

## Discussion

The first few minutes and hours immediately after birth are the most critical period in the life of a newborn. Hence, preventing neonatal morbidity and mortality requires equipping mothers with accurate knowledge of newborn care to ensure proper practices. In this study, only 33.5% of mothers had good knowledge of ENC. This may result in failure to achieve the national goal of reducing neonatal mortality from 28 to 11/1,000 by 2020 (20). This result has a prodigious inference on the survival of the neonates, opposing preventable disease, and future development and productivity.

The magnitude of good knowledge of ENC in this study is in line with prior studies from Ethiopia (10, 21) despite being lower than studies conducted in Nekemte city, Ethiopia (19, 22) and Ghana (23). This could be due to the study setting variation since this study was conducted in a rural area, a place where access to health facilities and health professionals is deprived and where traditional home practice is widely exercised. Additionally, poor quality postnatal services, counseling, and inadequate home visits could aggravate this issue. Mothers with poor knowledge of ENC are more likely to use harmful traditional practices which may affect the health and growth of newborns and create a negative impact on the current global and national plan to reduce neonatal mortality by 2030 (24).

The finding on the level of good knowledge in this study is also higher than the studies conducted in India (25) and Bangladesh (26). This might be due to the study population in India consisting only of primiparous mothers, while adolescent women (aged 15–19 years) who had ever been married with noninstitutional births were the study population for the study conducted in Bangladesh. Therefore, adolescents, primiparous mothers, and mothers who delivered at home might be less knowledgeable about ENC. Moreover, differences in the socio-cultural composition of the study groups and differences in the study period might be the reason for this discrepancy.

The care of the umbilical cord always needs special attention as it can function as the entry point for infections. Almost 26.1% of the respondents in this study practiced unsafe cord care and 34.9 % of them applied butter to the cord immediately after delivery. This is supported by findings from Ethiopia (19, 27). Locally, many people believe that applying butter, animal dung, or Vaseline would lubricate the cord and prevent dryness and cord and neonatal cloth attachment (21). However, this could result to be a source of infection which later complicates the survival of the neonate. In summary, this indicates that mothers had a lack of knowledge about appropriate cord care.

Regarding breastfeeding knowledge, 75.7% of mothers stated that breastfeeding should be initiated within 1 h. Although exclusive breastfeeding should be the only source of nutrition and energy for infants up to 6 months of age, only 59% of them reported that newborns should exclusively be breastfed until 6 months. On the other hand, WHO universally recommends that colostrum, the first milk that flows within the first few days after delivery, is the perfect food for every newborn, but only 39.8% of mothers feed colostrum to their newborn in this study. This is in line with a study conducted in southern Ethiopia (21), though incongruent to others (19, 22). This incongruity could be due to service delivery of information and demonstration about effective breastfeeding techniques for breastfeeding mothers or variation in health service utilization, culture, and taboos about breastfeeding (colostrum) within various areas. Early initiation of breastfeeding is encouraged as it stimulates breast milk production and facilitates the release of oxytocin, which helps in uterine contraction and reduces postpartum hemorrhage (28). Culturally, mothers milk out the first milk from the breast before attaching the newborn to the breast because they believe that the initial breast milk/colostrum is dirty and not nutritious. Therefore, they believe that the newborn must be supplemented with pre-lacteal feeds like butter and honey (29).

Hypothermia has significant contributions to neonatal morbidity and mortality in Ethiopia (30). According to this study, the thermal care practiced among respondents included initiating newborn bathing within 24 h after delivery (41%), using a cape to cover the head of the newborn (82.7%), and the exercise of skin-to-skin contact with the mother immediately after delivery (23.9%). Although the proportion varies, similar studies were being reported (10, 19). Strong cultural beliefs could be the main reason for common thermal care malpractices. The baby is seen as dirty after delivery, particularly if the vernix was visible, leading to the stigmatization of the mother. This is due to the belief that the vernix is the result of sperm secondary to late trimester sexual practice (31).

The presence of the routine provision of ANC had a significant impact on the mother's level of knowledge about ENC, which is consistent with studies done in Mekelle, Ethiopia (10), Kenya (32), and Ghana (23). Maternal and child health care are interrelated. The more women who adhere to the continuum of maternal and childcare, the more women who will know the benefits of care, understand health appointments, and improve their communication with care providers. Therefore, we recommend that the maternal and child health services, including ANC and PNC, should be further strengthened, especially at rural community levels.

Educated mothers were more likely to be knowledgeable about ENC. The role of maternal education as an important determinant factor of a mother's knowledge of ENC has also been reported by other studies (10, 22). Education leads to an increased level of awareness regarding maternal and neonatal illnesses, health-seeking behavior, and availability of services, which may sensitize the family to decide and utilize health care provided at various health care facilities (7). The Ethiopian government assigned HEWs in each kebeles to improve maternal and child health service delivery in the district by delivering appropriate information about the different available services to the mothers at the community and household level. But this alone does not seem to be enough to increase the level of maternal knowledge. The findings from this study also revealed that there is a significant association between mothers' religion and knowledge of ENC. A similar finding was reported in Uganda (33). This implies there is a need to further study with analytical study design for future researchers.

Institutional delivery is also significantly associated with ENC. Mothers who delivered at health institutions were about four times more likely to have good knowledge of ENC when compared to mothers who delivered at home. The result was in line with a previous study reported from Ghana (23). This might be because mothers who deliver at the health facility may get better counseling regarding ENC, PNC, and danger signs. Hence, they feel quite confident about their health and the health of their newborn. This indicates that home deliveries may be missed opportunities to promote ENC's benefits for mothers and newborns. In addition, the findings suggest that there is a need to improve the quality of maternal health care services (counseling or health education and promotion) at the rural community level.

### Strength and Limitation

Since this study was carried out at the community level in rural Ethiopia, it has the chance to gather the opinion of participants at the grass-roots level and helps to produce means to improve the services and knowledge gaps of ENC among the rural community. However, as a limitation, the study was focused on reported rather than observed knowledge levels of ENC. In addition, due to the cross-sectional nature of the study, it could be difficult to see the causal relationship between the independent and the outcome variable. Again, the study assumes homogeneity among the kebeles which may not be true in real circumstances.

## Conclusions

Based on this study, mothers' knowledge level of ENC was significantly low in the study area. Antenatal care visits, counseling during ANC place of delivery, delivery place, religion, and maternal educational status were factors significantly associated with the mother's knowledge of ENC. Cultural practices, such as tying the cord with unsterile materials, applying butter on the newborn cord, giving home remedies for cord infection, avoiding colostrum feeding, and home delivery practices, were common. Therefore, it is better to give special attention to ensuring that education should focus on women for antenatal care follow-up, encouraging institutional delivery, and inspiring counseling to rural and illiterate mothers.

## Data Availability Statement

The original contributions presented in the study are included in the article/Supplementary Material, further inquiries can be directed to the corresponding author/s.

## Ethics Statement

The studies involving human participants were reviewed and approved by Haramaya University, College of Health and Medical Sciences, Institutional Health Research Ethics Review Committee (IHRERC). The patients/participants provided their written informed consent to participate in this study.

## Author Contributions

TG: conceptualization, investigation, methodology, project administration, analysis, writing—original draft, and writing—review and editing. MD: conceptualization, methodology, writing—review, writing—review and editing, and supervision. AE: conceptualization, methodology, writing—original draft, and writing—review and editing. BB: investigation, methodology, writing—original draft, and writing—review and editing. TY: conceptualization, analysis, writing—review and editing, and supervision. The co-authors wrote the manuscript. All authors were involved in reading and approving the final manuscript.

## Conflict of Interest

The authors declare that the research was conducted in the absence of any commercial or financial relationships that could be construed as a potential conflict of interest.

## Publisher's Note

All claims expressed in this article are solely those of the authors and do not necessarily represent those of their affiliated organizations, or those of the publisher, the editors and the reviewers. Any product that may be evaluated in this article, or claim that may be made by its manufacturer, is not guaranteed or endorsed by the publisher.

## Abbreviations

ANC: antenatal care
CSA: central statistics agency
ENC: essential newborn care
HEWs: health extension workers
PNC: postnatal care
SDGs: sustainable development goals
WHO: World Health Organization.
