# Supplementary material for: Magnitude and Determinants of Postnatal Mothers' Knowledge of Essential Newborn Care at Home in Rural Ethiopia
Source: Front Pediatr. 2022 Apr 29;10:860094. doi: 10.3389/fped.2022.860094 (PMC9099071; doi:10.3389/fped.2022.860094)
Supplement: Supplementary file 1 [file Data_Sheet_1.PDF]

Questionnaire

English Version Questionnaire

Questionnaire ID no.________

General instructions

1.Choose one of the alternatives given for each question
2.If your answer is not found among the given alternative tell your answer to the interviewer

S. No_	Questions	Response	Code	skip
I.	Socio-demographic characteristics			
101	The age of your infant?	________		Next visit
102	What is the sex of your baby?	1. Male<br>2. Female		
103	How old are you?	_____ years		
104	Can you read and write?	1. Yes<br>2. No →		**106**
105	What is the highest level of education you attained?	1. No formal education<br>2. Primary (1-8$^{th)}$<br>3. Secondary (9th-12$^{th)}$<br>4. College and above		
106	What is your current marital status?	1. Single<br>2. Married →<br>3. Divorce/ separated<br>4. Widowed<br>5. Other (specify)____		**107**
107	What is educational status of your husband?	1. No formal education<br>2. Primary (1-8$^{th)}$<br>3. Secondary (9th-12$^{th)}$<br>4. College and above		
108	What is your religion?	1. Orthodox<br>2. Muslim<br>3. Protestant<br>4. Others (specify)____		
109	What is your ethnic group?	1. Oromo<br>2. Amhara<br>3. Tigre<br>4. Other (specify)_______		
110	Your occupational status	1. Governmental employee<br>2. Private employee<br>3. Housewife<br>4. Farmer<br>5. Daily laborer<br>6. Merchant<br>7. Student<br>8. Other (specify)_____		

111	Your residence?	1. Urban<br>2. Rural		
**Part II: Obstetric and maternal health service information**				
201	How many times did you gave birth?	________		
202	How many times you became pregnant (including abortion, stillbirth)?	________		
203	How many times do you experienced abortion?	________		
204	Did you attend ANC during your recent pregnancy?	1. Yes<br>2. No →		**209**
205	How many times have you attended ANC in your recent pregnancy?	1. One<br>2. Two<br>3. Three<br>4. Four and above		
206	Whom did you see during your recent ANC visit?	1. Physician<br>2. Health officer<br>3. Midwife/nurse<br>4. HEWS<br>5. Other(specify)___		
207	Did you receive counseling during your recent ANC period?	1. Yes<br>2. No →		**209**
208	About what did you counseled? (Multiple answer is possible)	1. Breast feeding<br>2. Maternal nutrition<br>3. Family planning<br>4. Hygiene<br>5. HIV awareness<br>6. Essential newborn care<br>7. Newborn danger signs<br>8. Immunization<br>9. Other(specify)____		
209	Where did you deliver your recent baby?	1. Hospital<br>2. Health center<br>3. Health post<br>4. Private health institution<br>5. Home<br>6. Other(specify)_____		
210	Who assisted you during your recent delivery?	1. Health professional<br>2. HEWs<br>3. TBA<br>4. TTBA<br>5. Relative(friend)<br>6. No one		
211	What was your mode of delivery?	1.Spontaneous vaginal delivery<br>2.Instrumental assisted delivery<br>3.Caesarean section		
212	Were you seen by health workers immediately after delivery?	1. Yes<br>2. No →		**301**
213	By whom did you seen?	1. Physician<br>2. Health officer		

		3. Midwife/nurse<br>4. HEWs<br>5. Other(specify)_____		
214	Did you receive counselling about neonatal danger signs during PNC?	1. Yes<br>2. No		
215	If yes to q-214, About what did you counseled? (Multiple answer is possible)	1.Breast feeding<br>2.Maternal nutrition<br>3.Family planning<br>4.Childbirth complications<br>5.Postnatal care<br>6.Essential newborn care<br>7.Newborn danger signs<br>8.Immunizations<br>8.Other(specify)____		

**Part III Maternal knowledge of Essential New born Care**			
**3.1 Breast Feeding**			
301	When is the appropriate time for initiation of Breastfeeding?	1. Within one hour<br>2. After one hour<br>3. don't know	
	Did you know that colostrum feeding is important for the new born?	1.Yes<br>2.No	
302	Have you squeezed out/removed the colostrum before you start breastfeeding?	1.Yes<br>2.No	
303	Did you feed your newborn the first milk (colostrum) that came out from your breasts?	1.Yes<br>2. No	
304	Have you provided anything other than breast milk for the new born?	1. Yes<br>2.No →	307
305	If yes for the above question, when did you give it for the first time?	_______	
306	What did you gave to the new born immediately after delivery? (Multiple answer is possible)	1.Nothing<br>2.Cow's milk<br>3.Plain water<br>4.Formula feed<br>5.Sugar water<br>8.Other (specify)	
307	Did you know that breast should be given on demand for the new born?	1.Yes<br>2.No	
308	Do you think that the newborn baby should be nursed in a room separated from his/her mother after delivery	1. Yes<br>2. No	
309	Did you now exclusive breast feeding is recommended for 6 months?	1.Yes<br>2.No	
**3.2. Immunization**			
310	Was your baby immunized at birth?	Yes<br>No	
311	Did you know that the new born need to be vaccinated at birth	Yes<br>No	

312	What is the importance of vaccination?	1.To prevent disease<br>2.I don't know<br>3.Other (specify)	
**3.3 Thermal care**			
313	Did you know about thermal care?	Yes<br>No	
314	What did you do to keep your baby warm? (Multiple answer is possible)	1.Skin to skin contact<br>2. Immediately wrapped the baby cloth<br>3.Cover head with a cape	
315	Which types of cloth you used to wrap the baby after bath?	1. New cloth<br>2. Clean and dry old cloth<br>3. Soiled and old cloth<br>8. Other, Specify______	
316	What is the correct time to bath your baby after delivery?	1.Within 24 hours<br>2.After 24 hours<br>8.Other(specify)	
317	Temperature of the water is?	1. Warm<br>2. Cold	
**3.4 Cord care**			
318	Do you know about cord care?	1. Yes<br>2.No	
319	What did you use to tie a cord?	1. Sterile cord tie<br>2.Unsterile material<br>3. I don't know<br>8. other (specify)__________	
320	Which instrument is used to cut the cord?	1. New blade<br>2. Used blade<br>3. Knife<br>4. Scissor<br>5. Other (specify)__<br>8. Do not know	
321	What did you do to the umbilical stump after the cord is cut?	1.Cover with cloth<br>2.Uncover, keep dry and clean<br>8.I do not know	
322	Did you apply anything on the stump after the baby's cord was cut?	1.Yes<br>2.No	
323	If yes, what was applied? (Multiple answer is possible)	1.Butter<br>2.Vaseline/oil<br>3. Antiseptics<br>4.Cow dung<br>5. Ash/soil<br>8.Other (specify)___	
324	What do you do if you see a sign of cord infection? (Multiple answer is possible)	1. Give home remedies<br>2. Delay until it heals by itself<br>3. Seek care from health facility<br>4. Other (specify)___	

Thank you for your cooperation!!
